# Career Barriers of Chinese Self-Expatriate Women: Neither Double Jeopardy nor Ethnic Prominence

**DOI:** 10.3389/fpsyg.2019.03024

**Published:** 2020-01-24

**Authors:** Nikos Bozionelos

**Affiliations:** EM Lyon Business School, Écully, France

**Keywords:** self-initiated expatriates, gender, careers, double jeopardy, ethnic prominence, Chinese, Caucasian, career barriers

## Abstract

The study explored barriers to career progression of Chinese self-initiated expatriate (SIE) women in the United Kingdom using semi-structured interviews with two matched samples of Chinese SIE and native Caucasian British women. Double jeopardy and ethnic prominence offered the theoretical backdrop. Common gender-related career hindering factors emerged in both groups. However, no barriers due to ethnicity *per se* emerged for the Chinese SIE women, whilst their narrations revealed that the Chinese stereotype had rather facilitated their careers in the host country. Furthermore, they did not view self-expatriation as a particularly challenging endeavor. None of the two interpretative frameworks deployed, double jeopardy and ethnic prominence, could account for the findings. The study implies that the effects of ethnicity on self-expatriation experiences and outcomes may be contingent on SIEs’ ethnic and cultural origins. In addition, the findings imply that women who self-expatriate to escape gendered opportunities in their home countries may face similar gender-related barriers in host countries.

## Introduction

The notion of self-initiated expatriation was introduced to signify those individuals who engage in expatriation, which is planned and initially intended as temporary move of their main residence to another country, purely through their own initiative and resources (Suutari and Brewster, 2000; Inkson and Myers, 2003; Jokinen et al., 2008). SIEs compose a very substantial population worldwide, outnumbering organizational expatriates by a ratio of approximately 20 to one (Finaccord, 2014)^1^. However, along with other forms of international mobility, only recently has systematic attention been directed to them (Doherty, 2013; Andresen et al., 2015; McNulty et al., 2017).

Another subject of interest in expatriation research has been gender. Available evidence on gender pertains mostly to organizational expatriation that has been studied for much longer (Doherty, 2013). Women are seriously disadvantaged in organizational expatriation, having around four times less chance than men to be sent abroad as organizational expatriates (e.g., Brookfield Global Relocation Services, 2015, 2017). This is alleged to be primarily due to gender-biased organizational decision-making that leads to discrimination against women in selection for organizational expatriation (Tharenou, 2010; Shortland and Perkins, 2019), organizational decision makers allegedly choose to overlook women as organizational expatriates (Shortland and Altman, 2011; Bohmer and Schinnenburg, 2016).

The difficulties women face in securing organizationally-assigned expatriation incentivize them to self-expatriate in order to gain international experience (Napier and Taylor, 2002; Tharenou, 2010). Similarly, women may choose to self-expatriate because they perceive career disadvantages in their home countries stemming from gender inequality in employment opportunities and gendered career ladders (Thang et al., 2002; Tharenou, 2010; Wechtler, 2018). Hence, they see self-expatriation as a means of finding a more equitable environment in the host country (Tharenou, 2010; Wechtler, 2018). Indeed, women are much more likely than men to self-initiate their expatriation (Andresen et al., 2015). The question then becomes what happens to women who self-expatriate. Does this pay off in terms of finding the opportunities they were hoping for and a more equitable and less discriminatory environment? Or do the disadvantages they feel they face in their home countries follow them as SIEs in the host countries? This is of substance to know, because if the disadvantage does not dissipate as result of the move abroad the implication is that the SIE route is not a career strategy that pays off for women.

SIEs allegedly face greater difficulties than their corporate-sponsored counterparts. This is because they do not have at their disposal the support of a home country corporation to deal with logistical, work and personal adaptation issues (e.g., Vance, 2005; Richardson, 2009; Fontinha et al., 2018), hence, they must rely exclusively on their own resources, material, physical and psychological, to succeed in the expatriate endeavor (Jannesari et al., 2017; Agha-Alikhani, 2018). The difficulties caused by self-expatriation itself, therefore, should augment the career difficulties SIE women may face in the work environment of the host country because of their gender. In addition, SIEs are more likely to find employment in domestic rather than multinational organizations in the host country (Jokinen et al., 2008; Peltokorpi and Froese, 2009), which may add to their difficulties given that international firms are presumably more accustomed and more sensitive to diversity (e.g., Clipa and Clipa, 2009; McDonnell and Scullion, 2013; Rofcanin et al., 2014; also Donnelly, 2014).

Women who move abroad are not a homogeneous group and distinctions must be made between those who belong to the dominant ethnic group of the host society and those who belong to ethnic minorities (Syed and Pio, 2010). Ethnic minorities, with particular reference to non-Caucasians in Western societies, face substantial barriers to their careers in ways similar to workplace and labor market discrimination women allegedly face (e.g., Stewart and Dixon, 2010; Alonso-Villar et al., 2012; Andriessen et al., 2012). To illustrate, evidence shows that non-Caucasian immigrants in predominantly Caucasian societies are disadvantaged in terms of job entry and in terms of occupational and financial attainment in comparison to their Caucasian immigrant counterparts (e.g., Reitz and Verma, 2004; Oreopoulos, 2011; Hosoda et al., 2012).

Ethnic minority groups are also likely to be over-represented among people who move from developing countries to developed Western countries as a response to generally limited opportunities in their home countries (Finaccord, 2014). It also appears that the career disadvantage for both women and ethnic minority groups is especially pronounced in highly skilled occupations and professions (e.g., see Singh, 2007), seen as occupations and professions typically performed by SIEs (Cerdin and Selmer, 2014). Professional and managerial careers have prestige and power attached to them, which means greater resistance from the dominant group (in the case of Western societies the dominant or reference group is Caucasian males) to grant power to minorities. As a consequence, the term “glass ceiling” has been coined (Morrison et al., 1987; Boone et al., 2013) to signify the barrier imposed on women’s and ethnic minority group members’ path to the top.

It follows from the above discussion that ethnic minority SIE women in developed Western countries should face additional barriers over and above the challenges imposed by the very nature of self-expatriation. It is, therefore, of interest and substance to find out what these barriers are and the impact they have on their careers. Such research will provide information that applies to a large proportion of the SIE population, given the prevalence of women and ethnic minorities within SIEs.

The present study aimed as an initial exploratory step in this direction by comparing career accounts of Chinese SIE women in the United Kingdom (UK) with career accounts of native Caucasian British women. The UK was highly suitable as host country setting because (a) it is a major developed economy with a predominantly Caucasian population and (b) it attracts large numbers of SIEs (Finaccord, 2014; Rienzo and Vargas-Silva, 2015; Fontinha et al., 2018). Using the approach of Tharenou (2010) and Finaccord (2014) to identify expatriates and SIEs, along with up-to-date definitions of SIEs (Andresen et al., 2014; Cerdin and Selmer, 2014; McNulty and Brewster, 2017) and the utilization of statistics of the British government (Office for National Statistics, 2019a, b) and other UK sources (Kone and Sumption, 2019) the estimate is that in 2019 there were approximately 1.1 million expatriates in the UK of whom approximately 1.04 million were SIEs. To account for the career experiences of the Chinese women, two competing arguments were deployed, double jeopardy and ethnic prominence. These arguments have been developed especially as frameworks to explain career experiences and outcomes of groups that represent cases of multiple minorities or disadvantage; hence, they are appropriate here.

### Double Jeopardy and Ethnic Prominence

#### Double Jeopardy

The double, or multiple, jeopardy argument was introduced by Beale (1970) and was further developed by other authors (e.g., Epstein, 1973; Almquist, 1975; Reid, 1984; King, 1988; also Hancock, 2007). It suggests that individuals who belong to more than one minority, subordinate or “stigmatized” groups are likely to encounter as many sources of discrimination as the minority or disadvantaged groups they belong to; and hence, they suffer added or even multiplicative negative effects (e.g., Settles, 2006). To illustrate, according to the double-jeopardy idea, non-Caucasian (e.g., African or East Asian origin) women who perform managerial or professional jobs in male-dominated, white societies are likely to face two sources of discrimination in the workplace and in the labor market: (1) discrimination because they are women; and (2) discrimination because they belong to an ethnic minority. In this respect, therefore, women who are SIEs and also belong to an ethnic minority may be facing disproportional challenges and barriers to their employment and career progression in the host country.

#### Ethnic Prominence

On the other hand, the ethnic prominence argument (Levin et al., 2002) posits that the dominant source of disadvantage due to discrimination is ethnicity, whose effects overshadow the problems associated with membership in other disadvantaged groups (Levin et al., 2002)^2^. The notion of ethnic prominence was developed specifically as an alternative to the double jeopardy with particular reference to being female as the second subordinate group. The reasoning behind the ethnic prominence view is that being an ethnic minority is more prominent and salient than gender, while at the same time ethnic minorities are also more likely to be viewed as threatening (Levin et al., 2002). To illustrate, according to the ethnic prominence view, women who belong to ethnic minorities will not experience greater disadvantage than their ethnic minority male counterparts, but both groups will experience handicap in comparison to the dominant ethnic majority. According to this premise, therefore, ethnic minority women in predominantly Caucasian societies, such as the UK, will experience workplace discrimination. However, this will not be because of their gender but only because of their ethnicity.

Though empirical work directly testing the two arguments is limited, extant research shows some support for both. Buchanan and Fitzgerald (2008) found a disproportional disadvantage for being both African American and female in documented cases of workplace harassment in the United States (US), which is in line with double jeopardy, and Syed and Pio (2010) found some evidence of double jeopardy in a study with career opportunities of first-generation Asian Muslim women in Australia. Two recent studies also provided some evidence for double jeopardy: Woodhams et al. (2015) in a study within a single firm in the UK found that pay decreased sharply along with increases in the number of minority groups employees belonged to, and Lavaysse et al. (2018) with a sample from the US workforce found a positive association between number of presumably disadvantaged groups the individual belonged to and perceived job insecurity. On the other hand, Levin et al. (2002) supplied some support for ethnic prominence by finding that women with African or Latin American origins in the US did not expect more gender discrimination than their male counterparts, but they expected to be discriminated against because of their ethnicity. In contrast, Caucasian women held expectations of discrimination because of their gender (Levin et al., 2002). Wolfram (2017) also found evidence for ethnic prominence in a study in the UK that utilized the probability of conjunction error (perceived over-representation of minority or disadvantaged groups in the workplace) as outcome: it was only being black, but not black woman that was associated with conjunction error.

It appears, therefore, that extant research on these two competing arguments is not conclusive about which holds truth. To account for the inconsistent findings, authors have suggested that both frameworks contain truth, but whether they are able to explain outcomes depends on contextual factors, such as the type of the job (Derous et al., 2012; Marcus and Fritzsche, 2015). This idea explains findings such as in Derous et al.’s (2012) multi-study work in the Netherlands, where both frameworks were supported depending on the condition: gender did not decrease the odds of job application success for Arab origin applicants in comparison to Caucasian Dutch applicants for relatively low status jobs, in line with ethnic prominence; but it did for the high status job condition, in line with double jeopardy. Nevertheless, Derous et al.’s design was not equivalent for the low and high-status job condition; hence, it is difficult to draw definite conclusions. Another factor that may be of relevance is ethnicity itself, and specifically the particular ethnic group to which the individual belongs. Both double jeopardy and ethnic prominence rely on the assumption that particular ethnic groups are viewed more negatively than the dominant ethnic group. However, there are differences in the degree of negativity (or positivity from the reverse angle) toward ethnic groups (e.g., Verkuyten and Zaremba, 2005; Priest et al., 2018). Consequently, the influence of ethnic origin on outcomes may vary across ethnicities – depending on the strength of the negative stereotype and *vice versa.* And that difference may impact whether double jeopardy or ethnic prominence applies to the particular situation. Hence, it is worth considering minorities that have been neglected in relevant empirical studies. In addition, it appears important to draw on both theoretical frameworks in a single study – unlike what most research has done so far – so their relative merits can be exploited.

### The Present Research

The aim of the present work was to conduct an initial exploration of perceived barriers in the careers of highly skilled Chinese SIE women in the UK using the rival views of double jeopardy and ethnic prominence as explanatory frameworks. For this purpose, the study examined the careers of managerial and professional Chinese SIE women in the UK and those of their native Caucasian British counterparts, who served as the reference group^3^. This methodology enabled first to find out whether the career barriers perceived by the Chinese SIE women would be different from those experienced by their native Caucasian British counterparts; and then to explore whether these differences could be accounted for by self-expatriation along with double jeopardy or ethnic prominence. This research contributes to the literature because:

(1) Research on the careers of SIEs is still limited. Women and ethnic minorities compose the largest part of that group in developed countries (see InterNations, 2018); therefore, it is essential to investigate the extent to which the burden of self-initiated expatriation is further encumbered by being both female and ethnic minority member in the host country.

(2) Extant research on self-initiated expatriation has neglected participants from Confucian countries like China, and has instead over-focused on SIEs from Western European and English-speaking countries (e.g., Cao et al., 2013; Muir et al., 2014; Vaiman et al., 2015; Makkonen, 2016; Lindsay et al., 2019; cf., Kemp and Rickett, 2018). There are substantial differences in cultural values between Confucian and Anglo-Saxon or Western European societies (Gupta et al., 2002), but also arguably in the way Caucasian “westerners” are treated in non-western non-Caucasian societies (Mathur-Helm, 2002), which may influence the way self-initiated expatriation is perceived and managed. This means that findings from studies with SIEs from developed Caucasian Western countries who find themselves in non-Western societies can by no means be generalized with certainty to SIEs from developing Confucian countries in developed Caucasian societies. Considering that SIEs from China compose a substantial part of the global SIE population (Finaccord, 2014), empirical research with Chinese SIEs in Western societies is necessary to allow safer conclusions on their experiences.

(3) Research on double jeopardy and ethnic prominence is still limited, and even more so of research that utilizes them together as theoretical frameworks. In addition, and perhaps most importantly, extant research has focused on ethnic minorities (Black, Latin American, Central-South Asian, such as Indian, Pakistani, Bangladesh, or Arab) whose stereotypes in Caucasian-dominated societies cannot be considered positive by any means (e.g., Derous et al., 2012; Pew Research Center, 2018; Priest et al., 2018)^4^. On the other hand, the stereotype of East Asians (i.e., Chinese, Japanese, and Koreans) is argued to be considerably more positive (Bobo, 2001; Clow and Esses, 2007; Priest et al., 2018). For example, Zhang (2015) found the stereotype of the Chinese was strongly linked with industriousness, high morals, inventiveness, and intelligence, and similar findings were reported by Bowler and Segura (2011). This may be partly because, as consequence of the Protestant work ethic, hard work is a central value in many Caucasian-dominated societies (especially North America, Australia and Western Europe, including the UK) (e.g., Van Hoorn and Maseland, 2013), which meets work as a core virtue of the Confucian value system that characterizes East Asian Societies (e.g., Hofstede and Bond, 1988). It will be of benefit, therefore, to investigate perceptions of female Chinese SIEs (part of the East Asian cluster) regarding their career experiences and barriers in host countries through the prism of double jeopardy and ethnic prominence. That will provide an initial idea of whether belonging to an ethnic minority is also associated with negative career experiences for SIEs of Chinese origin. Considering the salience of ethnicity in today’s social and business environment with strong international mobility, this is subject worth looking at. Finally, extant studies with double jeopardy or ethnic prominence have focused on single events or outcomes, such as finding or being shortlisted for a job. In contrast, the present study looked at overall careers in the host country. Because careers are built as the “evolving sequence of a person’s work experiences over time” (Arthur et al., 1989, p. 8) they are shaped by the cumulative effect of numerous work-related events and the experiences these generate (Greenhaus et al., 2000). Hence, careers are more likely to reveal the effects of disadvantaged groups membership than a single occasion or events of a single class.

Factors that may influence careers in general and careers of women and ethnic minorities in particular pertain to human capital (for example, recognition of qualifications in the host country, psychological resources), social capital (for example, connections or lack of connections with others who can help one’s career), structural factors (e.g., the type of the industry, administrative barriers), work-family interference, and stereotyping, the implicit belief that people with particular demographics fit or do not fit particular roles (Baruch and Bozionelos, 2011; Hirschi, 2012; Legrand et al., 2019). Hence, career barriers were considered within the framework provided by that set of factors.

The employment relationship has been changing over the past decades, moving from normative (as in most part of the previous century) toward less standardized employment relations based on individualized deals and more fragile ties between employees and employers (e.g., Kalleberg and Marsden, 2015). This change has been parallel (partly causing and partly being the cause) to globalization, a manifestation of which is the movement of labor, such as SIEs, across national borders (O’Donohue et al., 2018). The way gender and ethnicity impacts work and career experiences of expatriates must have implications for their motivation, performance and intentions to stay in the host country, and it is therefore important for all stakeholders: SIEs themselves, their host country employers, and host countries.

The research questions were as follows: What are the career barriers Chinese SIE women face in a predominantly Caucasian society such as the UK? Does their ethnicity and self-expatriation cause barriers beyond those caused by their gender? Are the dominant intersectionality arguments of double jeopardy and ethnic prominence able to account for their career barriers?

## Materials And Methods

A qualitative interpretivist approach was adopted because this allows the discovery of facts that may not have been foreseen by the researcher (Ghauri and Gronhaug, 2010; Hennink et al., 2011). This approach takes into account the fact that individuals assign their own meanings to events and situations, hence, people’s accounts need to be studied in depth in order to develop a thorough understanding of the phenomenon under consideration (Crotty, 1998). Levin et al. (2002) imply that different ethnic origins may influence discrimination and barriers in qualitatively different ways, hence, their discovery clearly necessitates qualitative research designs.

Data were collected via semi-structured interviews, all conducted in English, from two groups: Ten Chinese SIE women and 10 native Caucasian British women in the UK. A biographical/life history approach was adopted to extract information on participants’ own perceptions of factors that played a central role in their careers (Cochran, 1990; Veale and Gold, 1998).

### Participants

The native Caucasian female group was necessary to employ in order to strengthen confidence that any identified influence of ethnicity and of self-expatriation would be validly isolated from the influence of gender. The Chinese SIE women were recruited first because they composed what was seen as the core group, but also because these were more difficult to find. Personal contacts were utilized to identify and approach individuals who fit the required profile: Chinese women employed full-time in professional or managerial occupations who had come to the UK independently and not as part of a corporate assignment. The author utilized his own personal contacts with Chinese people who were working in the UK and also solicited acquaintances to provide him with contact details of Chinese people in the UK whom they knew personally. Initial contact with these potential participants was made in order to: (a) screen whether the person fitted the criteria of SIE and (b) ascertain whether the individual would be willing to participate. This process was preferred from the frequently utilized snowballing technique for validity reasons. Though the snowballing technique may have facilitated the process it would most likely have resulted in a more homogeneous sample (people tend to associate with others who have similar experiences and attitudes to them, Byrne, 1971) that would restrict the variance in experiences and perceptions and, hence, would lead to biased findings (Lee, 1993; Hearn et al., 2008; Shortland and Altman, 2011). An ethics approval was not required for this study as per applicable institutional and national guidelines and regulations. Oral informed consent was obtained from all participants.

Participants met virtually every articulated criterion that relates to SIE characterization (Doherty et al., 2013; Andresen et al., 2014; Cerdin and Selmer, 2014; McNulty and Brewster, 2017): (a) their initial intention but also their expectation at the time of the interviews was for a temporary move; (b) they had legally moved and were legally residing and working in the host country; (c) the move to the host country was on their own free will rather than critical push factors (that can be, for example, civil war, persecution due to political beliefs, famine); (d) the decision about their employment was made exclusively by the host country employer, and they had initiated and executed their move themselves without assistance from the home country. Participants also met a criterion that some authors consider relevant to SIE status (McNulty and Brewster, 2017), none had acquired the home country (in this case British) citizenship. Finally, although it was not one of the required features, it also emerged that none of the Chinese SIEs had any international work experience before arriving in the UK. Chinese self-expatriate interviewees were added until saturation with respect to ethnicity-related factors had been reached. Saturation was reached with the analysis of the eighth interview, in line with evidence that saturation in research that uses existing theoretical frameworks is normally reached within 6–12 interviews (e.g., Guest et al., 2006).

The native Caucasian British women were selected so that their professional and demographic profiles matched, to the extent possible, those of the Chinese women. The process was that for every Chinese female participant, a British woman of similar personal and work (i.e., occupation, industry) demographics was sought. The demographics of participants are presented in Table 1. Mean age for the British and Chinese participants was 38.22 (*SD* = 8.02, *range* = 29–50) and 40.11 (*SD* = 6.19, *range* = 33–51) years, respectively. The average time in the UK for the Chinese group was 6.39 years (*SD* = 1.19, *range* = 5–8.5). That meant they had spent a substantial amount of time in the UK; hence, they had accumulated significant experience to allow the development of a representative picture of barriers and other career issues they had faced.

**TABLE 1:** Demographics of participants.

**Name (Pseudo)**	**Age**	**Employment**	**M status/children**	**Years in the UK**	**UK Region**
**Chinese women SIEs**					
Hwei-ru	38	Law firm (lawyer)	Married/one	7	South Yorkshire
Li	42	Law firm (lawyer)	Married	6	Durham
Guan-yin	51	Hospital (Admin)	Married/one	8.5	Tyne and Wear
Lee	37	Physician (Hospital)	Single	5	North Yorkshire
Qing	48	Physician (Hospital)	Married/two	7.5	Nottinghamshire
Nuo	42	Banking (Analyst)	Married/two	7	Greater Manchester
Mingxia	33	Banking (Analyst)	Single	5	London
Shun	36	Broadcasting (assistant producer)	Married/one	5.5	Yorkshire
Xia	34	Restaurant (manager)	Married	6	Leicestershire
Yue	40	Retail chain (manager) Single	Single	6	Durham

**Name (Pseudo)**		**Age**	**Employment**	**M status/children**	**UK Region**

**Native caucasian british women**				
Linda		43	Law firm (lawyer)	Married/two	South Yorkshire
Jane		40	Police (lawyer)	Married	Tyne & Wear
Fiona		50	Construction firm (admin)	Married/one	Tyne & Wear
Judith		35	Physician (Hospital)	Married/two	North Yorkshire
Anne		50	Physician (Hospital)	Married/one	North Yorkshire
Lindsay		34	Banking (Analyst)	Single	Greater Manchester
Alison		29	Banking (Analyst)	Married/one	London
Catherine		32	Recruitment (Consultant)	Married/two	Yorkshire
Bridget		31	Nursery (Manager)	Single	Staffordshire
Tracy		38	Hotel Chain (Manager)	Single	Durham

### Interview Content and Procedure

Interview duration varied from 45 min to 1 h and 45 min. Anonymity and confidentiality were assured. At the beginning, participants were asked to narrate their career histories to date in any ways they considered appropriate, and to highlight factors that were barriers and enhancers to these. The Chinese women were asked to focus on their employment and careers since they arrived in the UK. However, they were also asked to briefly talk about their experiences in their country of origin. Once the end of the career narration was reached, questions were asked on how participants thought their careers were going to develop in the future and whether they perceived any barriers, past and future, toward their career progression. During the whole process, prompting was utilized for further explanation or clarification when the researcher considered necessary. This format allowed for the discovery of facts as perceived by interviewees, but it also conferred adequate control on the researcher (Howitt, 2010).

### Analysis

Though there were specific interpretive frameworks, the researcher had to be fully aware of the possibility that the particular theoretical frameworks may be unable to exhaustively account for the data (Ghauri and Gronhaug, 2010; Howitt, 2010). Therefore, the researcher kept an open mind in case the data would direct toward findings that did not fit the theoretical models. This preparedness proved to be essential, because – as will be seen below – neither double jeopardy nor ethnic prominence provided a satisfactory account of the data.

The analysis was conducted manually (i.e., without specialist software). Scripts from the interviews were analyzed using the following themes as guide: human capital (e.g., education, work experience, language capacity, cultural factors, personality), social capital (e.g., network ties), structural factors (e.g., firm size, male vs. female dominance in the firm/organization, occupation or industry), work-family interference (e.g., roles at home that may clash with career priorities), and stereotyping. These themes follow theorizing on factors that influence career advancement in general as well as women’s and ethnic minorities’ in particular (Baruch and Bozionelos, 2011; Hirschi, 2012; Legrand et al., 2019). Following recommendations on thematic analysis (Krippendorf, 2004), interview scripts were searched for information that pertained to these themes and could be interpreted in light of women’s and ethnic minority’s career advancement. In the first stage, transcribed interviews were searched and the material was coded according to each of these five themes. The next stage involved sub-coding according to specific aspects within each theme. The coded information was then considered against career obstacles or enhancers pertinent to gender, to ethnicity, and to the move to the host country (the latter two for the Chinese women only). With respect to gender, narrations of the two groups of women, native Caucasian British and Chinese SIEs, were compared by looking at the content of their responses in the gender domain (i.e., codes and sub-codes that were related to gender). This enabled to identify both the nature of the career barrier (gender vs. non-gender-related), but also the specific source (e.g., stereotyping, social capital, etc.).

There were a number of reasons to believe that credibility, the extent to which the researcher’s interpretation of reality based on participants’ accounts actually match what participants really experienced and meant (Lincoln and Guba, 1985), was established in the study. In line with guidelines (Lincoln and Guba, 1985; Tracy, 2010; Korstjens and Moser, 2018), not all interviews were conducted by the same person (some were conducted by the author and some others by an assistant), “negative” cases (i.e., the minority of cases where perceptions of interviewees were different from the perceptions of the majority) were reflected upon, and – maybe most important – the researcher kept a neutral stance in the analysis. As noted earlier, the findings with respect to Chinese women’s experiences contrasted the expectations of the researcher, which is strongly supportive of the credibility of the study.

## Findings

### Gender-Related Career Barriers

Most participants, nine Chinese and eight British, perceived barriers to their careers because of their gender. Perceptions of gender barriers were mostly related to direct discrimination in the workplace connected with stereotyping, including stereotyping about the type of work women can do and stereotyping about family-to-work interference (i.e., women are not fit for professional or managerial careers and/or they are not able to dedicate themselves to the employer because of family obligations). For example, the British lawyer who was working for the police stated: “Women are not considered competitive in the police. Even when you are proved as brilliant as or even better than your male colleagues, people still question your ability and tend to attribute your success on other things.” [Linda, British, age 44, two children]^5^; and Li [Chinese, age 42, married, no children] noted: “I have no children … chance to have I’d say none … still I sense the thinking of partners and the rest …I can’t give myself one hundred percent to the firm.” It is worth noting that the discrimination was not only viewed as coming from within the workplace but also from clients. One of the participants (an analyst in investment banking) noted: “When my male colleague and I go to meet clients together, clients are more likely to listen to my male colleague’s advice instead of me. As they still think that, as a woman, I may not be able to give good investment consulting as my male colleague. [Lindsay, British, age 34, single, no children].

Overt gender discrimination was more likely to be felt by the women working in male-dominated industries (e.g., banking) regardless of their ethnic origin than in industries where power is more balanced between the two sexes (such as broadcasting and the nursery). For example, a Chinese lawyer noted: “My boss has given me plenty of encouragement and support. But I cannot see my future in this firm because you look at it, the top people are all men!” [Hwei-ru, Chinese, age 38, married, one child]. Hence, structural factors (type of industry or male-dominance in the particular firm) seemed to be a parameter in perceptions of gender discrimination as a barrier to advancement. Nevertheless, participants considered that being female was a disadvantage even in apparently female-dominated industries or firms. For example, the native British participant who was working as recruitment consultant felt that: “As recruitment is stereotyped as a job for women, few male counterparts enter that job. Even though, male’s promotional opportunities are still higher than women’s,” and added “men somehow make it feel natural they get higher faster,” and justified the cause of it as “deep-rooted belief that the firm is secure when men are in the saddle.” [Catherine, Chinese, age 32, two children].

There was only a small minority of women (three out of twenty) who did not feel gender-related discrimination in their workplaces. One of them, a physician working for a hospital, pointed out: “As long as you don’t self-characterize yourself, you would find people respect you and accept you the same as your male co-workers.” [Judith, British, age 35, married, two children]. Nevertheless, even these women considered that they had to compromise their involvement with their families in order to achieve as much as their male counterparts.

Human capital, in terms of qualifications, skills and knowledge, was not an issue for any participant. The only human capital factor that emerged as relevant to career progression was personality (for a categorization of human capital, see Baruch and Bozionelos, 2011). Most women perceived that they had to conform to a masculine personality stereotype, including assertiveness and not expressing emotions, in order to achieve success: “Police work … you get to see a lot even if you are not in the street or in the lab…to show emotion does not mean you are weak… but this is what everybody thinks for a woman.” [Linda, British, age 43, married, two children]; and Xia [British, age 34, married, no children] noted: “I don’t know what you know about the restaurant business, but believe you me it’s tough…the kitchen and everything… that’s why chefs are always men…a woman doesn’t look like a man to survive has to act like two men” [Xia, 34, married, no children].

Social capital did not emerge as a factor directly, but did so indirectly in terms of workplace male dominance. According to all participants, British and Chinese, this inevitably put women at a disadvantage in forming ties with important others because those others were in most cases men. For example, Xia [Chinese, age 34, married, no children] noted: “all key matters are with men… to get power for your interests you must be close to men”. They considered that building networks was still feasible, but they had to invest more time and effort than their male counterparts did. For example, Nuo [Chinese, age 42, married, two children] noted: “First, you must go near men with force… you don’t know how others see that, a woman near men all the time …,” and “for men it comes as is to hang around with men”. Hwei-ru [Chinese, age 38, married, one child] mentioned:

Men partners and associates go for drinks after work… for our firm it translates seven or eight in the night … I am invited … I could go if my husband looks after our son … can be arranged no joke, but how often?

Alison [British, age 29, married, one child] noted:

[it] Has to come from us women, to do our power networking our way, organize events the way it fits us, like the French revolution… nobody will disallow, but it can’t be done overnight … many women think it is all right as it is.

It clearly emerged, therefore, that gender-related issues were seen as barriers to career progression by both groups of women, British and Chinese SIEs.

### Career Barriers Due to Ethnicity

Beyond factors that pertained to gender, it was very difficult to discern in the narrations and answers of Chinese participants any career barriers attributions to their ethnic origin *per se*. Only one Chinese perceived barriers because of discrimination caused directly by her ethnicity. That individual (lawyer working for a law firm) reported problems of social inclusion in her workplace:

I am excluded in the workplace. I have few British friends, and I will not have opportunities to be promoted to partner level. For British women, if you work as hard as your white male colleagues, you would be noticed and promoted. But not for us foreigners. [Li, Chinese, age 42, married, no children]

That participant also expressed the intention to return to China at some point soon. The only human capital factor that clearly emerged as a career barrier exclusively to Chinese women was English language proficiency (imperfect accent included). This, according to all Chinese participants, could create communication problems, which could in turn negatively impact social capital by reducing their ability to form close ties in the workplace. For instance, two Chinese women mentioned difficulties in developing rapport with native British colleagues because of mutual difficulties in understanding each other’s jokes. In addition, they believed that language proficiency could initially cause issues with perceptions of competence. For example, one Chinese participant noted that at the beginning, some others doubted her ability:

At the beginning, people wondered if I can actually do the job as my job requires lots of communications, either with people inside of the company and from outside. They doubted about my English skills and my understanding of doing things in this country. [Shun, Chinese, age 36, married, one child]

Another human capital factor that was seen as a hindrance to career prospects by a minority (two) of Chinese women was a low-key behavioral profile [“modest, soft, caring and supportive,” as one Chinese participant (Lee, age 37, single) worded it] that is associated with appropriate female behavior in China. This, according to them, was an impediment to work and advancement opportunities because their supervisors tended to value pro-activeness and self-promotion rather than a humble, low-profile style.

It should be noted that, even when prompted, none of the Chinese women mentioned any serious problems with the acceptance of their qualifications. This included the six participants who completed all their education in China. This contrasts with the frequently reported problems with recognition of credentials or suspicions regarding the quality of home country education for people who move to host countries (Suto, 2009). An explanation may be that the participants who had completed all their education in China had already secured employment in the UK before their actual physical move to the UK.

Social capital, and especially a perceived difficulty to break into the male or female networks of the native British, was seen as a problem by three Chinese women. Reasons for this, as expressed by participants, have already been mentioned earlier and included difficulties communicating because of language or because of cultural differences (e.g., perceptions of humor, philosophy of doing business). Nevertheless, most (seven) perceived no more difficulties in breaking into influential networks than their female British counterparts did. Only one Chinese woman perceived social exclusion simply because she was not British (i.e., overt discrimination) – Li, Chinese, age 42, married, no children, whose case was mentioned earlier. In all other cases, Chinese women linked their social capital problems with linguistic and cultural issues rather than the mere fact of their ethnicity.

Finally, most (eight) Chinese women perceived that the quality of their work was appreciated, and native British were prepared to accommodate them when they proved their worth. For example: “When I proved that I could do the job, people started to accept the fact that I am no different than them.” [Shun, Chinese, age 36, married, one child].

### An Unexpected Finding: The Perceived Positive Effect of Being Chinese

An unexpected, interesting and potentially important finding that emerged from the narrations of all Chinese women was the perception that Chinese ethnicity offered mostly advantages. Through their experience in the UK, Chinese participants had formed the impression that the stereotypical image of the Chinese contained features such as industriousness, self-discipline and perseverance. Because of that reason, according to them, employers and line managers were positively predisposed and inclined to trust them and assign them responsibilities. The following narrations are illustrative: “My employer prefers to assign me important jobs because he says that I proved myself trustful, disciplined and industrious, just as his expectations from a Chinese.” [Yue, age 40, single, no children]. And “I knew of the image of us [the Chinese] … kind soon the manager put me in a project that was…special I would say … you are Chinese [he said] … no matter what it takes you’ll give them what they want when they want” [Nuo, age 42, married, two children]. And Lee [physician, age 37, single, no children] noted: “The consultant … had said … we are overworked here, but you are Chinese we have no worries.”

In fact, it emerged that the advantage of that stereotype was so strong that in some cases it was seen as counter-balancing the disadvantage of being female. Indeed, in the narrations of four Chinese it was evident that they thought this stereotype was so beneficial that it neutralized the negative impact of gender on their career prospects. To illustrate, Nuo [banking analyst, age 42, married, two children] noted: “sometimes it feels that foreigner, better say Chinese, is not as I expected a load but an asset … maybe it’s the place or my job but that Chinese I feel turns my chances better than other women’s here”. And Shun [age 33, married, one child] noted: “(smiling) either China is too strong or the Chinese are too good workers!. either reason … I’m given chances.”

### Challenges of Self-Initiated Expatriation

Self-expatriation itself was not viewed as a particular challenge by the Chinese women. There was one exception, the participant [Li, Chinese, age 42, married, no children] who also perceived ethnic discrimination. Most of the married participants (four out of seven) were already married when they had arrived to the UK; hence, they did not face the logistical issues of the movement alone, despite having no support from a home company. However, those who were not married at their arrival did not perceive any noteworthy difficulties either. When prompted for additional explanations for the relatively trouble-free move to the UK, many (six) noted that they had had to endure moves across substantial physical distances when they were in China (to study and afterward to join their employer) or other kinds of separation and “hardship.” It appeared that this experience had familiarized them with the issues to face when moving to another country. Some of them also mentioned the “friendliness” of the British State and of the British “system.” The account of Hwei-ru [age 38, married, one child] is illustrative:

I lived separate from my parents since…forever. I stayed with my grandparents because my parents were working in [name of Chinse city] hours away. I saw them only occasionally and not for long… only in new year for that. I joined them in the last years of secondary school that was a school with good reputation in the city they worked. Then in the university I lived alone … graduated and started working, my company sent me for six months in Inner Mongolia, thousands kilometres from [name of Chinse city]. I married and moved with my husband to [name of major Chinese city] … also long distance and very different from my home place… another move makes no much difference. HR helps and officials are friendly. Supermarkets are the same everywhere! I’m financially secure, nobody forced me to come here, I have a good life.”

Mingxia’s [age 33, single, no children] account is also interesting:

“From very young I saw my parents … they had put me internal in a reputable school in another city…only in week-ends and summers. After university, in [name of major Chinese city], I stayed there because I found work in a good firm. In a training event I met someone from my company here who told me about the opportunity. I wanted to know things abroad and was good for the cv. They helped with the move, I am happy here, there are Chinese I can meet if I want. I chat any time with my friends in China. Everyone complaints about the weather, but I don’t sweat like in South China and when it’s cold we have heating! My parents visited me, but it is my granny I feel close… she raised me. Taking the British nationality I have to give up my Chinese that will cause many problems back. Anyway, I guess I will return, maybe in a couple of years.”

At this point, it is also worth noting that in their narrations, the Chinese women appeared generally more determined than their British counterparts in dealing with problems that may come their way, including gender discrimination and family responsibilities. For example, they indicated greater preparedness to compromise time with their family. To illustrate, Nuo [Chinese, age 42, two children] noted:

After I gave birth to my second son, the partners wondered if I could still commit to my career as before. But when they see me working until late at night, just as before, they still assign important jobs to me. but I really feel guilty to my kids. Whenever I am able, I will take them with me. [Nuo, age 42, two children]

The above implies that cultural features may be at play in the way the Chinese SIE women perceived and managed their careers in the host country.

## Discussion

In essence, the findings were compatible with neither of the invoked frameworks. With respect to double jeopardy, Chinese women perceived some difficulties resulting from not being native British, and these were over and above the hardships caused by their gender. However, these difficulties could by no means be characterized as workplace discrimination because of ethnicity *per se*. The issues reported were related to linguistic and cultural factors that sometimes hindered communication and accumulation of social capital. These causes, however, certainly do not identify with direct ethnic discrimination, which is the idea behind the double jeopardy argument. Prejudice because of different use of language, including accent, is common even between native speakers of the same ethnic groups who simply reside in different regions of the same country (Skutnabb-Kangas and Phillipson, 1989). Direct discrimination would involve, for example, natives being negatively biased simply because of non-native accent or natives consciously avoiding social interaction without any regard for that person’s credentials, performance or other qualities (e.g., Dietz, 2010; Hosoda et al., 2012). However, the Chinese participants did not report or hinted something like that. Equally important, such issues dissipated once the Chinese women proved their competence, which again argues against ethnic discrimination.

The fact that ethnicity was not perceived as cause of problems with employment and career progression automatically rendered the argument of ethnic prominence unable to provide an account either. In fact, the findings attest to the existence of an effect that one may label “reverse ethnic prominence”; in that having Chinese ethnicity was often regarded by participants as an advantage, sometimes perceived as able to counterbalance the negative effects of being female. That was an unexpected and apparently significant finding. Chinese participants attributed the advantageous functioning of their ethnicity to the stereotypical image of the Chinese, with features that are valued in the work and business environment, such as industriousness, self-discipline and perseverance. According to their accounts, that image created a positive bias that brought benefits. It is important to note here that nearly all (nine out of 10) Chinese SIEs were located outside London. This strengthens the findings in the sense that diversity is much lower outside the “superdiverse” London area (Natham and Lee, 2013), and lower diversity may make ethnic discrimination more likely. The finding was unexpected, but it may nevertheless look less odd in light of recent studies that find that on average Caucasians in the US perceive East Asians as more hardworking and equally intelligent to Caucasians themselves (Bowler and Segura, 2011), or they would rate East Asians more highly than Caucasians, even in scenarios where Asians were depicted as immigrants (Visalvanich, 2017).

It should also be noted that these findings do not appear to fit even the alternate and more recently proposed theoretical framework of intersectional invisibility (Purdie-Vaughns and Eibach, 2008). That model was introduced especially to account for cases that neither double jeopardy nor ethnic prominence is able to satisfactorily explain, and proposes that multiple subordinate group membership reduces the probabilities of being noticed. This can ensue either positive (e.g., lower probabilities of being spotted when a failure has taken place) or negative outcomes (i.e., less probability to be noticed or commended when a positive act has occurred), depending on the situation. In the present study, however, Chinese female SIEs did not emerge as “invisible,” but as the opposite instead. Their accounts indicated that they were visible both as women, with negative perceived consequences, and especially as Chinese, with positive perceived consequences, and these were the reasons behind many of their career experiences.

A noteworthy implication of this study, however limited in scope it is, therefore, is that ethnic origin should not be treated as a unitary factor (i.e., that its effects are uniform across ethnicities). The findings clearly imply that certain ethnic origins may offer advantages, instead of disadvantages, in self-expatriation (or in other endeavors of similar nature). This is a possibility that appears to have been overlooked thus far. These findings indicate a type of interaction between being female and having Chinese ethnicity that contrasts extant frameworks, and implies that the way multiple group membership affects experiences and outcomes may be more complex than has been generally assumed.

With respect to self-initiated expatriation, at odds with what has been assumed so far, it emerged that the Chinese women did not view it as particularly demanding and challenging endeavor. There are two, not mutually exclusive, explanations. First, as they reported themselves, the Chinese participants had already experienced separation along with movement across locations, albeit within their native country. That may have accustomed them with dealing with separation and relocation on their own – which is part of the SIE experience. Second, dominant cultural values in Confucian societies, such as the Chinese, are perseverance and resilience (e.g., Hofstede and Bond, 1988). These may have endowed them with attitudes that enabled them to both overcome and assimilate most difficulties they faced.

What emerged about self-expatriation raises the issue of SIEs’ country and ethnic origin in the experience and outcomes of that endeavor. In recent years, and as work on self-initiated expatriation grows in volume, authors have looked at various factors to explain SIEs’ adaptation and success in host countries (e.g., Bozionelos, 2009; Cao et al., 2013; Selmer et al., 2015; Husssain and Deery, 2018; Lauring and Selmer, 2018). However, the ethnicity and the country of origin of the SIE has not been openly discussed or empirically considered thus far. The findings of the present study imply that ethnic origin may play a substantial role in SIEs’ adaptation and success in the host country. Furthermore, empirical research on self-expatriation has so far been dominated by Western samples. The present study implies that some assumptions drawn with those samples may not necessarily hold for all SIEs and how self-expatriation is experienced and managed.

The findings also make implications about whether women who self-expatriate to find career opportunities they cannot find in their home countries really benefit from the move in that respect (e.g., Tharenou, 2010; Wechtler, 2018). What can be deducted from the findings is that, at least according to the accounts of the Chinese SIEs themselves, self-expatriation does not remove the career barriers posed by gender. Indeed, the accounts of the Chinese SIE women indicated that they were exposed to similar gender-related issues and situations as their native host country female counterparts. On the other hand, the findings imply that women from particular ethnicities may enjoy career benefits in expatriation provided they move to host countries where the stereotype of their ethnicity is positive.

The substantial increase in international mobility has made career management more complicated for both internationally mobile workers themselves, but also for host country employers and host countries alike. In terms of demographics, women in earlier times had to develop career strategies to minimize the gender barrier. In the present era, however, concerns for the many women who self-expatriate may also include ethnicity and how this is perceived in the host country. The way SIEs are treated, or perceive they are treated, in the host country workplace and the host-country in general, should have serious implications for their adjustment, performance and input for host country employers and the host country overall (Husssain and Deery, 2018). Being aware of how self-expatriate women, who compose a very substantial in size group, experience self-expatriation as function of their gender and ethnic origin is, therefore, of importance for host country employers, managers and officials.

### Limitations and Directions

It is important to keep in mind that the study was limited in scope, hence, it should be seen as an early investigation whose findings open interesting possibilities for future research on a subject of substance. In addition, the study recorded the perceptions of actors, and there is no guarantee that those perceptions were accurate representations of reality. For example, it does not necessarily mean that because participants perceived that they were discriminated against because of their gender actual discrimination was taking place. From the inverse viewpoint, there is a possibility, however limited, that there was in fact workplace discrimination taking place against the Chinese participants but they were not consciously aware of it. Nevertheless, there is good reason to believe that at least the expressed views of participants were genuine. For example, the two individuals who conducted the interviews were not native British but foreigners instead (one female Chinese and one non-British male). Being interviewed by non-natives should have removed reservations for the Chinese participants and made it more likely that they would describe ethnicity-related negative experiences, but they rarely mentioned such experiences. Following from the above, future research should also seek the views and experiences of colleagues and superiors of these SIEs. This will enable triangulation and will provide information on how others within the workplace view the SIEs (also Shortland and Altman, 2011).

The employment profile of participants was restricted to high-profile occupations and professions. Though this was a conscious choice (according to some authors professional qualifications/skills is one of the conditions for characterization of someone as SIE, Cerdin and Selmer, 2014), it raises the issue of whether the findings would be similar with Chinese who pursue operational-level, lower-skill jobs. Such jobs, and the individuals who perform them may be viewed of lower importance by employers. The UK apparently has a chronic shortage of people who perform complex highly-skilled jobs (e.g., Migration Advisory Committee [MAC], 2019). Hence, employers may particularly value foreign workers who are able to perform such jobs well, and this may have been partly responsible for the experiences of the Chinese SIE women in the present study.

The fact that what was analyzed was perceptions (women’s perceptions of career disadvantage) permitted using females only in the study. However, a more complete account with respect to the ethnic prominence idea could be obtained with the consideration of the experiences of male Chinese SIEs as well. Ethnic minority males may be more likely to experience discrimination and disadvantage than their female counterparts because they are the minority males who the dominant group (typically the males of the dominant ethnic group) perceive as threatening (Sidanius and Pratto, 1999). Therefore, it will be important to find out whether the apparent beneficial effects of Chinese ethnicity for female SIEs applies to their male counterparts too.

Finally, the findings imply that SIEs may represent a group with particular characteristics with respect to personality and motivational features. This may be one of the topics for future SIE research. For instance, the narratives and life stance of the SIE women in the study imply that they possessed strong self-efficacy, resilience and optimism, which are constituent elements of psychological capital that has been connected with enhanced career outcomes (Luthans et al., 2015). Future studies may investigate the role of psychological capital in self-expatriation outcomes, and compare the psychological capital of successful SIEs with individuals in other forms of international mobility and with individuals who made the choice not to self-expatriate or who returned prematurely.

## Data Availability Statement

The datasets for this article are not publicly available in order to preserve participant anonymity. Requests to access the datasets should be directed to NB, bozionelos@em-lyon.com.

## Ethics Statement

An ethics approval was not required for this study as per applicable institutional and national guidelines and regulations. Oral informed consent was obtained from all participants.

## Author Contributions

The author confirms being the sole contributor of this work and has approved it for publication.

## Conflict of Interest

The author declares that the research was conducted in the absence of any commercial or financial relationships that could be construed as a potential conflict of interest.

## Footnotes

1 Specifically, the number of expatriates, individuals who had legally moved and were legally living temporarily (without permanent residence rights or citizenship) to a host country (i.e., their primary address was in the host country) was estimated at 207.6 million worldwide in 2013 (Finaccord, 2014). Of those, 6.9 million were organizational expatriates (“corporate transferees,” Finaccord, 2014). These numbers exclude refugees and asylum seekers (Finaccord, 2014). These estimates, therefore, bring the ratio of SIEs to organizational expatriates to 29 to 1 worldwide. If only those individuals who had legally moved and were living and working (as opposed, for example, to students, trailing spouses, retirees, etc.) temporarily in the host country – conditions that meet closer recent definitions of the SIE, Andresen et al., 2014; Cerdin and Selmer, 2014; McNulty and Brewster, 2017) – are considered the number of SIEs worldwide in 2013 is brought to 130 million (which brings the ratio of SIEs to organizational expatriates to 19 to 1).

2 In the present work “ethnicity” is treated as tantamount to “race.” Though these are different constructs, our Chinese participants were all racially and ethnically identical: they all displayed the same distinct physical characteristics (“race”), and they all possessed the characteristics of Chinese ethnicity given that they had been borne, brought up, lived the largest parts of their adult lives in China and identified themselves as “Chinese” (ethnicity).

3 The term “native Caucasian British” is utilized to indicate that all British women participants were born in the United Kingdom (UK) and were Caucasian, which has traditionally been the dominant ethnic group in the UK.

4 It is reminded that a stereotype is a simple and overgeneralized view about characteristics of members of social groups (Hamilton and Trolier, 1986) or “a widely held but fixed and oversimplified image or idea of a particular type of person or thing” (Oxford Dictionaries, Def. 1).

5 Interviewees’ narrations are quoted verbatim and unaltered, regardless of any grammatical imperfections they may contain.
